# Eosinophilic esophagitis in children: doubts and future perspectives

**DOI:** 10.1186/s12967-019-2014-0

**Published:** 2019-08-09

**Authors:** Elena Cavalli, Andrea Brusaferro, Elena Sofia Pieri, Rita Cozzali, Edoardo Farinelli, Gian Luigi de’ Angelis, Susanna Esposito

**Affiliations:** 1 Pediatric Clinic, Department of Surgical and Biomedical Sciences, Università degli Studi di Perugia, Perugia, Italy; 2 Gastroenterology Unit, Department of Medicine and Surgery, University of Parma, Parma, Italy

**Keywords:** Autoimmunity, Dysphagia, Eosinophilic esophagitis, Food allergy, Monoclonal antibody, Esophageal symptoms

## Abstract

**Background:**

Eosinophilic esophagitis (EoE) is a chronic immune-mediated inflammatory disorder and represents the leading cause of food impaction. The pathogenesis of EoE is the result of an interplay between genetic, environmental and host immune system factors. New therapeutic approaches for EoE have been proposed. In this manuscript we review the current evidence regarding EoE management in pediatric age, with a particular focus on new findings related to the efficacy and safety of monoclonal antibodies.

**Main body:**

Conventional therapies have failed in treating some patients with EoE, which then requires aggressive procedures such as esophageal dilatation. The most effective available medical therapy for EoE is swallowed topic corticosteroids (fluticasone propionate and budesonide), which have two main drawbacks: they are related to well-known adverse effects (especially in the paediatric population), and there are not enough long-term data to confirm that they are able to reverse the remodelling process of the esophageal mucosa, which is the major cause of EoE symptoms (including dysphagia, abdominal pain, nausea, obstruction, perforation and vomiting). The monoclonal antibodies appear to be an interesting therapeutic approach. However, the studies conducted until now have shown substantial histological improvement not coupled with significant clinical improvements and no significant relationship between a decreasing number of eosinophils and clinical symptoms, highlighting the importance in the pathogenesis of EoE of cells such as T-helper cells, mast cells, B cells, epithelial cells and natural killer cells.

**Conclusions:**

Monoclonal antibodies targeting a signal involved in the pathogenesis of EoE may not break the complex self-propagating inflammatory activation responsible for perpetuation of the inflammatory response and the development of symptoms and complications. We speculate that combined biological therapies targeting more than one molecule or cell may provide better results, with conventional therapies potentially enhancing the effects of antibodies. However, further studies should aim to find the best therapeutic approach to target the cells involved in the remodelling process and to reverse the histological changes in this complex clinical condition.

## Background

Eosinophilic esophagitis (EoE) is a chronic immune-mediated inflammatory disorder, usually considered part of the wide spectrum of food allergies defined symptomatically by esophageal dysfunction and histologically by eosinophil-predominant inflammation of the esophagus [1]. It is the leading cause of food impaction, the main cause of dysphagia among children and young adults in Europe and North America and the most prevalent cause of chronic or recurrent esophageal symptoms after gastroesophageal reflux disease [2].

EoE has evolved from a rare case-reportable condition to a disease that is commonly encountered in the clinic and endoscopy suite [3] and a major cause of upper gastrointestinal morbidity and increasing health care costs [4]. In children undergoing gastroscopy, irrespective of the cause, the prevalence was 3.7% [5, 6]. In the general population, it is estimated to be about 30–52 cases per 100,000 inhabitants [5, 6]. The incidence and prevalence of EoE are rising at rates that outpace increased recognition [5–7]: this increase is therefore not just an artefact of increasing surveillance and detection [8]. These data seem to indicate the importance of environmental changes, rather than genetics, in the increased number of cases [9, 10]. EoE may occur at any age with a mean age at the time of diagnosis ranges between 5.4 and 9.6 years in children and a peak in adults at 30–35 years [1].

New therapeutic approaches for EoE have been proposed. In this narrative manuscript we review the current evidence regarding EoE management in children, with a particular focus on new findings related to the efficacy and safety of monoclonal antibodies. The MEDLINE and PubMed databases were searched for all of the studies published over the last 15 years using the key words “eosinophilic esophagitis” and “children” or “pediatric” or “paediatric”. Only articles published in English were considered and discussed.

## Pathogenesis

The pathogenesis of EoE is the result of an interplay between genetic, environmental and host immune system factors. The first event is the introduction, through ingested food, of antigenic proteins to the esophagus: this event, in genetically susceptible subjects, triggers a prevalent T-helper type 2 (Th2) inflammatory response [11], producing large amounts of Th2 cytokines, including interleukin (IL)-4, IL-5 and IL-13.

IL-4 is known to induce naïve T cells into Th2 cells and to activate B cell class switching to produce IgE, thus initiating a Th2-mediated immune response [11]. IL-5 is one of the main mediators of EoE, as it induces eosinophil production and eosinophil trafficking to the esophagus [12]. This cytokine has a narrow set of cellular targets, as in humans, only eosinophils, basophils and a subset of mast cells are known to express the IL-5receptor-alpha (IL-5Rα; CD125) chain [13]. IL-13 produced by Th2 cells and activated eosinophils induces esophageal epithelial cells to secrete eotaxin-3, the other main mediator of EoE, which recruits and drives eosinophils and mast cells from the peripheral blood into the tissue [14]. IL-5 and IL-9 produced by eosinophils enhance the growth and survival of eosinophils and mast cells, leading to a self-propagating cascade of eosinophilia and mastocytosis. Activated eosinophils and mast cells produce pro-fibrotic factors such as transforming growth factor (TGF)-β1 and fibroblast growth factor (FGF)-9, causing remodelling changes of the epithelium and subepithelium responsible for the characteristic symptoms and complications of EoE [15].

It is worth noting that in some cases EoE appears to be triggered not only by food but also by aeroallergens, although in a part of the cases no clear trigger can be identified [2, 8, 9].

## Clinical features

In younger children and infants, the most common symptoms reported are reflux-like symptoms, vomiting, abdominal pain, food refusal, and failure to thrive. Older children and adults with EoE most commonly report solid food dysphagia, food impaction, and non-swallowing—associated chest pain [16]. Although not associated with mortality or risk of malignancy, the chronic and progressive nature of EoE and associated symptoms negatively impacts the quality of life of patients [17].

The natural history of EoE consists of chronic inflammation that may progress into fibrous remodelling of the esophageal wall, with collagen deposition, fibrosis of the lamina propria, and development of esophageal strictures and narrow-calibre oesophagus, as the disease evolves from childhood into adulthood [18–20]. These histological changes reflect changes in the symptomatology.

Symptom-focused outcome studies have indicated a relatively benign course of the disease with absent or only mild dysphagia in most patients, with or without use of medical or dietary therapy directed at EoE. Several years after a diagnosis of EoE, approximately 30–50% of children transitioning to adulthood reported symptoms of dysphagia. However, patients tend to change their eating behaviours, avoiding specific food textures (meat, bread), increasing the use of liquids with meals and increasing mastication, which could contribute to the reduction in the occurrence of dysphagia from esophageal strictures. For this reason, the use of symptom assessments alone as instruments of diagnosis or monitoring of the disease is not recommended [10]. Moreover, dysphagia might be a dynamic symptom, as fibrous remodelling and its effects on the formation of esophageal strictures may change dysphagia over time, from an intermittent muscular phenomenon to a constant obstructive rigidity [21].

EoE has been recently recognized as a transmural disease in which the eosinophilic infiltration permeates deep into the submucosa, the muscle layers and the neuronal plexus, which could explain the disconnection between symptoms and the biological activity of EoE [11]. In contrast to symptom-based studies, studies focusing on endoscopic outcomes have reported the progression of significant fibrostenosis in most patients with over a decade of untreated EoE [19, 22–24].

## Diagnosis

EoE can be diagnosed by upper gastrointestinal endoscopy, taking at least six biopsies from different locations in the esophagus, focusing on areas with endoscopic mucosal abnormalities, in order to increase the diagnostic sensitivity. Biopsies should be taken even in cases of a normal endoscopic appearance of the esophagus, as this has been reported in up to 32% of children with EoE [25]. The accepted threshold for eosinophil density for the diagnosis of EoE is 15 eosinophils per high-power field (eos/hpf) in at least one esophageal mucosal biopsy, taken as the peak concentration in the specimens examined [26–29]. Other histological markers may include eosinophil microabscesses, basal zone hyperplasia, dilated intercellular spaces, eosinophil surface layering, papillary elongation and fibrosis of the lamina propria [30].

Non-invasive biomarkers, such as total IgE, eosinophil-derived neurotoxin, mast-cell tryptase, chemokines, and fractionated exhaled nitric oxide, have all failed in the diagnosis or monitoring of the disease [31–33].

## Treatment

EoE is a chronic disease with frequent progression to strictures and a narrow-calibre esophagus, which indicates the need for treatment. Such therapy should aim not only for the resolution of clinical symptoms but also for the resolution of esophageal inflammation, in order to obtain mucosal healing, which would allow the avoidance of long-term complications related to subepithelial fibrosis and deterioration in health-related quality of life. Another important therapeutic goal is to prevent adverse effects and complications related to long-term therapy and to avoid nutritional deficiencies derived from dietary restriction, especially among paediatric populations [2].

### Dietary therapy

Dietary therapy constitutes the only treatment targeting the cause of the disease: its goal is to identify and consequently exclude foods that trigger and maintain the disease from the diet [34]. Elemental diets outperform all other dietary-based and most drug-based strategies available in terms of inducing histological remissions of EoE [35], but their use in clinical practice is inhibited by several disadvantages.

The palatability of the elemental formula is indeed poor and led to naso-gastric tube placement in 80% of the children in one of the largest studies in which it was tried [36]. Such a restrictive and monotonous diet also has an enormous impact on a patient’s psychological well-being and social life [37, 38]. Moreover, the elemental diet is expensive and could delay speech onset in young children as a result of undeveloped facial muscles from an exclusive liquid diet [39, 40]. In addition, it could have an impact on taste development and delay the acquisition of feeding skills [41]. This means that this diet could then be considered for children who have severe symptoms and are refractory to other therapies or as a short-term approach to attempt to induce remission more rapidly [39, 42].

The empiric six-food elimination diet consists of avoiding foods that are most associated with food allergy (cow’s milk protein, wheat, egg, soy, peanut, fish/seafood). Kagalwalla et al. [43] demonstrated histological remission in the majority of patients (74%) treated with this therapy. Sequential reintroduction of foods followed by endoscopy and biopsies could identify in a minority of cases the specific food triggering EoE in each patient. However, this approach is invasive, as it requires sequential endoscopies. Spergel et al. [44] showed that an elimination diet based on skin prick test and atopy patch test results led to resolution of esophageal eosinophilia in a similar proportion of patients as empiric removal of foods but required that fewer foods be removed.

A step-up approach consists of eliminating one or two foods (milk and gluten) that are more related to food allergy and increasing the restriction only in non-responders. Molina-Infante [45] studied this approach in adult and paediatric populations, demonstrating that approximately half of the patients (43%) achieved clinical-histological remission. Kagalwalla [46] demonstrated that the percentage of remission increases to 64% in children treated with a four-food elimination diet (milk, egg, wheat, soy).

### Corticosteroid therapy

Steroids have been used as treatment for EoE from the initial description of the disease in the literature [47]. Initially, systemic steroids were employed to achieve clinical-histological remission. However, several studies demonstrated that topical steroids lead to the same results, avoiding the adverse effects of systemic administration [48, 49]. For this reason, systemic steroids are no longer recommended for EoE [1], except in emergency situations with severe dysphagia or significant weight loss [2].

Swallowed topic corticosteroids are superior to placebo and non-steroid therapy in decreasing eosinophil density in the esophageal mucosa [50, 51] and in achieving clinical remission [52]. The two molecules utilized are fluticasone propionate and budesonide: these molecules can usually be found as preparations for bronchial or intranasal delivery, which must be swallowed. A particular budesonide-based preparation, named oral viscous budesonide, has proved superior to fluticasone propionate, and this could be explained not only by its intrinsic anti-inflammatory properties but also by the more prolonged contact between the mucosa and the medication due to the sucralose utilized in its preparation [35].

Swallowed topical steroids seem to be safe; the few adverse effects are superficial esophageal candidiasis (described in up to 10% of patients; it responds to specific treatment) and rarely adrenal suppression and growth impairment [53]. Therefore, children with EoE should undergo cortisol monitoring [1].

Studies showed that the combination of topical steroids with diet elimination is not superior to single treatment in achieving clinical or histological remission. However, combination therapy could be effective in patients who have previously failed single-agent therapy [54].

### Proton pump inhibitor (PPI) therapy

In past years, PPIs had been considered a diagnostic tool to identify PPI-responsive esophageal eosinophilia, a term that referred to patients who initially appeared to have EoE but who obtain complete remission after PPI therapy [55–58].

PPI therapy is now considered a first-line therapeutic alternative to topical steroids and the elimination diet. Lansoprazole and rabeprazole show the highest efficacies in inducing histological remission, but there is a limited number of studies comparing the different molecules [59]. No significant differences were shown with a double daily administration compared with a single dose.

Patients who have esophageal eosinophilia and esophageal symptoms that resolve with PPI therapy have phenotypic, molecular, mechanistic, and therapeutic features indistinguishable from similar patients who do not respond to PPIs. In these patients PPI responsiveness is documented by reduction in Th2 inflammation and reverse in the abnormal gene expression signature [60].

Discontinuation of PPI therapy typically leads to symptomatic relapse. Therefore, PPI therapy should be used to maintain remission over the long term in patients with an initial response, along with a progressive decrease in dosage to the lowest dosage that keeps the disease in remission [1]. Long-term use of PPI has been shown to be safe in adults; however, no studies in children are available [61].

### Biological therapy

**Fig. 1:**
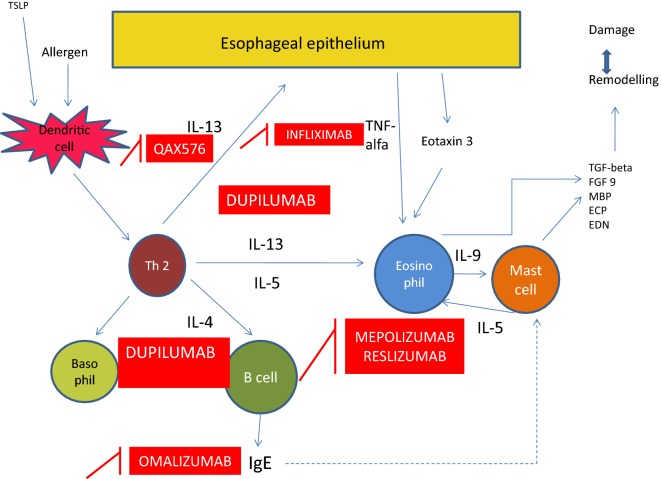
Mechanism of action of monoclonal antibodies for eosinophilic esophagitis (EoE) treatment. *ECP* eosinophilic cationic protein, *EDN* eosinophil-derived neurotoxin, *IgE* immunoglobulin E, *FGF* fibroblast growth factor, *MBP* major basic protein, *TGF* transforming growth factor, *Th2* T-helper type 2, *TNF* tumour necrosis factor, *TSLP* thymic stromal lymphoprotein

Monoclonal antibodies specifically targeting inflammatory effectors involved in EoE pathogenesis are studied to offer more potent relief of histological and clinical features, decreasing adverse effects to a minimum. Table 1 summarizes the main studies performed with the use of monoclonal antibodies for EoE treatment and Fig. 1 shows their mechanism of action.

**Table 1:** Summary of clinical studies on monoclonal antibodies for eosinophilic esophagitis (EoE) treatment

Study	Design	Drug	Target	Population
Assa’ad et al. [70]	Randomized double-blind parallel group	Mepolizumab	IL-5	Children (n = 59)
Otani et al. [71]	Randomized double-blind single arm	Mepolizumab	IL-5	Children (n = 43)
Spergel et al. [72]	Randomized double-blind, placebo control	Reslizumab	IL-5	Children (n = 226)
Arasi et al. [77]	Case report	Omalizumab	IgE	Child (n = 1)
Rocha et al. [73]	Case series	Omalizumab	IgE	Children (n = 2)
Loizou et al. [79]	Non-randomized, open-label, single arm	Omalizumab	IgE	Children and adults (n = 15)
Clayton et al. [78]	Randomized double-blind, placebo control	Omalizumab	IgE	Children and adults (n = 30)
Rothemberg et al. [62]	Randomized double-blind, placebo control	QAX576	IL-13	Adults (n = 25)
Straumann et al. [85]	Non-randomized, open-label, single arm	Infliximab	TNF-alpha	Adults (n = 3)

*IL* interleukin, *TNF* tumor necrosis factor

#### Monoclonal antibody anti-IL-13

Rothenberg’s randomized placebo-controlled study, conducted on a population of young adults, demonstrated that QAX576, a fully human anti-IL-13 mAb, significantly reduced esophageal intraepithelial eosinophils (up to 60% of baseline levels versus 23% in the placebo group) and improved the expression of a set of genes that are demonstrated to have a role in the pathogenesis of EoE, particularly the already cited eotaxin-3, periostin and markers of mast cells and barrier function. These results, however, were not accompanied by a clear improvement in symptoms [62], which could be explained by the fact that the present investigation was based on a small and heterogeneous population composed of patients refractory to conventional therapies. An important finding of this study was that the modifications caused by QAX576, both in terms of the number of intraepithelial eosinophils and in terms of gene expression, were still present 6 months after the administration of the last dose. For this reason, the authors argued for a role of this molecule as an additive with other therapeutics. The most important adverse effects of this drug are cough and gastro-esophageal reflux. Other anti-IL-13 drugs are currently under evaluation [1].

#### Monoclonal antibody anti-IL-5

IL-5 has an important role in the pathogenesis of EoE: in confirmation of this role, it has been demonstrated to be overexpressed in the esophagus of patients with EoE [63, 64] and to correlate positively with eosinophil levels and disease activity [65]. Moreover, overexpression of IL-5 can induce EoE in mice [12, 66, 67], and mice deficient in IL-5 are protected from EoE [68, 69].

Two types of antibodies have been developed to target eosinophils: antibodies against IL-5 (mepolizumab and reslizumab) and an antibody against IL-5 receptor (R)α (benralizumab). The first two antibodies bind to IL-5, interfering with the occupation of the IL-5R, while the latter antibody binds to the membrane-expressed receptor; in both cases, inhibition of signalling and induction of cell lysis are achieved [13].

In Assa’ad’s [70] study on mepolizumab, children obtained a significant reduction in esophageal eosinophilia, which was still observed at week 24; in particular, up to 89.56% of patients reached a mean esophageal eosinophil count under 20/HPF. Mepolizumab showed disappearance of eosinophilic microabscesses for at least 16 weeks after the last dose with an increase in tissue eosinophilia. Moreover, there was no significant clinical improvement.

Otani’s double blind randomized study on children with EoE who underwent treatment with mepolizumab highlighted the role of mast cells in the pathogenesis of this pathology. Indeed, anti-IL-5 therapy with mepolizumab lowered the number of eosinophils, which resulted in a decrease in the number of mast cells as well. The study also showed a relationship between reduction in mast cell numbers and improvement of symptoms; moreover, in a subset of patients, there was a direct correlation between the number of mast cells and the severity of some symptoms, such as stomach pain. In contrast, there was no correlation between eosinophil numbers and clinical manifestations [71].

In a randomized double-blind trial, Spergel [72] obtained a significant improvement in the peak esophageal eosinophil count in patients treated with reslizumab (median reduction up to 64% in patients treated with a high dose of reslizumab compared to a reduction of 24% in the placebo group). However, this histological response was not related to a clinical improvement, which was obtained both in the reslizumab and placebo groups.

In summary, although treatment responders displayed endoscopic improvements, substantial clinical benefits were not obtained. This could be related to the fact that the mepolizumab study enrolled symptom-free patients with no power to detect improvements, and in the reslizumab trial, improvements were also seen in the placebo arm. The disappointing clinical response to anti-IL-5 in these trials could be explained by the perpetuation of disease activity sustained by the residual tissue eosinophils and by the brevity of these trials, which could not document the reversal of fibrosis and remodelling. Overall, anti-IL5 antibodies were well tolerated, with the most common adverse effects being abdominal pain, diarrhoea and vomiting.

#### Monoclonal antibody anti-IgE

Omalizumab is a humanized monoclonal antibody that targets the high-affinity receptor binding site on human IgE, preventing the activation of mast cells [73, 74]. This drug is currently approved in several countries for the treatment of severe asthma and chronic urticaria and has been tried as an off-label treatment in other diseases, such as EoE [75, 76].

The first trial of omalizumab in EoE was in the case of a 13-year-old boy with severe EoE who experienced clinical remission with no persistent clinical, endoscopic or histological improvements [77]. In a two-case report, a girl and a boy received omalizumab, again with no persistent clinical and histological improvement [73]. Clayton’s randomized double-blind placebo-controlled study on adults and children showed mast cell IgE depletion after treatment with omalizumab, with no significant histological and clinical improvements [78].

On the contrary, Loizou’s [79] open-label single-arm trial demonstrated endoscopic and histological remission (peak esophageal eosinophil count < 15/hpf) in 33% of the subjects enrolled, which was more pronounced in children than in adults. Clinical remission was achieved in one-third of the subjects, while an improvement of symptoms was achieved in 47% of patients. Interestingly, EoE remission was obtained in patients with low peripheral-blood absolute eosinophil counts. Esophageal mast cell reduction was seen after treatment but was not associated with a reduction in oesophageal eosinophil counts. This study showed a high drop-out rate, but patients who completed the trial did not have serious adverse effects.

The lack of improving EoE by blocking IgE with monoclonal antibodies could be explained by the recent discoveries that a mixed immune response is observed in EoE and the role of IgG4 should still be elucidated. Dense infiltration by IgG4-positive plasma cells has been observed around the vessels of the lamina propria of adult EoE patients [80].

#### Monoclonal antibody anti-TNFα

Although it is generally accepted that EoE pathogenesis is related to a Th2-inflammatory response, Th1 cytokines such as tumour necrosis factor (TNF)-α and interferon (INF)-γ are found in increased quantity in the esophageal mucosal biopsies of EoE patients [81]. TNF-α produced by dendritic cells may be ultimately responsible for the remodelling process, which leads to esophageal strictures in so far as it induces the epithelial cells to contract, migrate and secrete collagen and enhances the expression of adhesion molecules on endothelial cells, leading to increased angiogenesis in the esophageal mucosa [82–84]. For this reason, infliximab, a chimeric IgG1 monoclonal antibody that inhibits TNF-α, was studied in a series of EoE patients. Straumann [85] conducted a pilot study on three adults refractory to conventional therapies. Even if the monoclonal antibody anti-TNF-α was well tolerated with no relevant adverse events, it did not show a significant decrease in esophageal eosinophil counts and did not improve clinical symptoms.

## New strategies

New molecules are studied for EoE treatment. Some of them target eosinophils, such as benralizumab which has already been used for severe asthma [86, 87], vedolizumab (an antibody that blocks the α4β7 integrin expressed on the eosinophil surface) [88], CCR3 antagonists, and Siglec8 antibodies. Dupilumab, an IL4/IL13 receptor antagonist used for atopic dermatitis, targets molecules along the Th2 axis and [89]. An encouraging pilot study with dupilumab in EoE has been performed and a large phase 3 study on its efficacy and safety in adolescents and adults with EoE is ongoing (clinicalTrials.gov identifier: NCT03633617). Other antibodies target thymic stromal lymphopoietin, which induces the production of Th2 cytokines [90, 91], and RPC4046, an antibody that binds to IL-13 receptors [92]. However, no definitive conclusions can be drawn on these new strategies due to the limited amount of data.

## Conclusions

Although the incidence and prevalence of EoE are rising, the optimal therapeutic approach for this disease has not been defined. Some patients fail to respond to conventional dietary and medical therapies, and subsequently require aggressive procedures such as esophageal dilatation. The most effective available medical therapy for EoE is swallowed topic corticosteroids (fluticasone propionate and budesonide), which have two main drawbacks: they are related to well-known adverse effects (especially in the paediatric population), and there are not enough long-term data to confirm that they are able to reverse the remodelling process of the esophageal mucosa, which is the major cause of EoE symptoms (including dysphagia, abdominal pain, nausea, obstruction, perforation and vomiting).

Although there are no biological therapies approved for EoE treatment, the monoclonal antibodies appear to be an interesting therapeutic approach. However, the studies conducted until now have shown substantial histological improvement not coupled with significant clinical improvements and no significant relationship between a decreasing number of eosinophils and clinical symptoms, highlighting the importance in the pathogenesis of EoE of cells such as T-helper cells, mast cells, B cells, epithelial cells and natural killer cells. Role of monoclonal antibodies targeting a single molecule (e.g., IL-13, IL-5, IgE, TNF-α) involved in the pathogenesis of EoE should be clarified, although these drugs may not break the complex self-propagating inflammatory activation responsible for perpetuation of the inflammatory response and the development of symptoms and complications. We speculate that combined biological therapies targeting more than one molecule and/or cell may provide better results, with conventional therapies potentially enhancing the effects of antibodies. However, further studies should aim to find the best therapeutic approach to target the cells involved in the remodelling process and to reverse the histological changes in this complex clinical condition.

## Data Availability

The data and materials used are included in the review.

## Abbreviations

ECP: eosinophilic cationic protein
EDN: eosinophil-derived neurotoxin
EoE: eosinophilic oesophagitis
FGF: fibroblast growth factor
IgE: immunoglobulin E
IL: interleukin
MBP: major basic protein
PPI: proton pump inhibitor
TGF: transforming growth factor
Th: T helper
TNF: tumor necrosis factor
TSLP: thymic stromal lymphoprotein
